# The Effects of Bio‐Fertilizer Applications on Post‐Harvest Fruit Quality in Soilless‐Grown Tomatoes

**DOI:** 10.1002/fsn3.72075

**Published:** 2026-07-01

**Authors:** Sedanur Yılmaz, Andaç Kutay Saka, Aslıhan Çilingir Tütüncü, Mehtap Özbakır Özer, Harun Özer

**Affiliations:** ^1^ Department of Horticulture, Faculty of Agriculture Ondokuz Mayis University Samsun Türkiye; ^2^ Department of Horticulture, Faculty of Agriculture Ordu University Ordu Türkiye; ^3^ Department of Horticulture Ministry of Agriculture of the Republic of Türkiye, Black Sea Agricultural Research Institute Samsun Türkiye

**Keywords:** *Arthrobacter globiformis*, *Aspergillus oryzae*, cold storage, *Streptomyces griseus*

## Abstract

Tomatoes ( *Solanum lycopersicum* ) are widely cultivated for nutrition and economic importance; quality losses of 25%–45% during cold storage pose a significant problem. In soilless cultivation, bio‐fertilization using beneficial microorganisms can extend shelf life by enhancing nutrient uptake and physiological resilience. This study investigated the efficacy of bacterial (AG: *Arthrobacter globiformis* ; SG: *Streptomyces griseus* ) and fungal (AO: *Aspergillus oryzae*) bio‐fertilizers applications on cluster tomatoes (Cletego F1) grown in soilless culture and during cold storage. Using a randomized complete block design with three replications, fruits harvested at the pink‐ripe stage were stored in Modified Atmosphere Packaging (MAP) at 8°C and 90% humidity for 21 days. Weight loss, respiration, firmness, pH, titratable acidity, SSC, vitamin C, phenolic/flavonoid content, and antioxidant activity were measured on days 0, 7, 14, and 21. Weight loss was highest in AO (0.11%) and lowest in AG (0.07%) on day 7. Respiration was highest in control (5.96 mL CO_2_ kg^−1^ h^−1^) and lowest in AG (3.58 mL CO_2_ kg^−1^ h^−1^) on day 21; AG (2.14%) maintained higher firmness than control (1.90%). SSC peaked in control on day 7 (5.90%), while microbial treatments reduced this value; vitamin C was highest in AG on day 21 (23.60 mg 100 g^−1^). AG showed highest values for phenolics (16.43 mg GAE 100 g^−1^) and flavonoids (13.57 mg QE 100 g^−1^) on day 21. DPPH was highest in AO (11.72), FRAP highest in control (20.04). Microbial fertilization, particularly with AG, supports nutritional quality by slowing respiration and enhancing bioactive compounds, while AO and SG influence antioxidant capacity.

## Graphical abstract

Microbial fertilization has been shown to have the potential to limit water loss. AG and AO treatments preserved shelf life by keeping the fruit flesh firmer compared to the control. Microbial fertilization preserved the physical structure of tomatoes during cold storage. Microbial fertilization activates the antioxidant defense systems of tomatoes during cold storage, thus delaying nutritional value loss. AG application improved the nutritional quality of tomatoes.

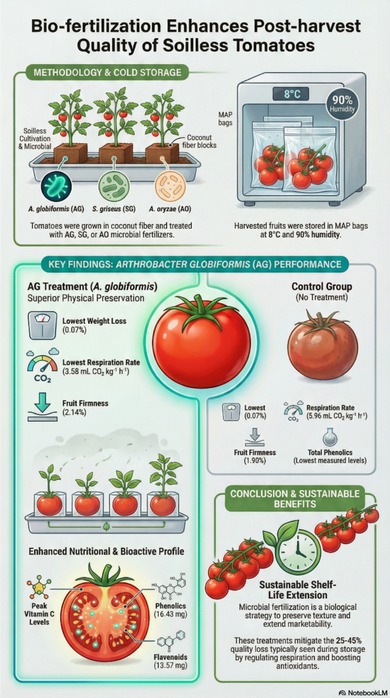

## 1 Introduction

The tomato is a globally significant vegetable crop, valued for its high nutritional content, including vitamins A, B, and C, as well as lycopene and flavonoids (Konar 2008; Tzortzakis and Economakis 2008; Gojiya et al. 2022; FAO 2024). However, traditional soil‐based tomato cultivation faces major challenges, including soil‐borne diseases, increasing salinity, and declining soil fertility, which often lead to excessive chemical use and environmental degradation. To mitigate these impacts, soilless cultivation systems have gained prominence over the last two decades, offering precise control over nutrient supply and higher resource efficiency (Sezen et al. 2010; Khan et al. 2021; Bwambale et al. 2023; Yılmaz et al. 2025).

In these systems, the plant root zone can be optimized by introducing beneficial microorganisms such as plant growth‐promoting rhizobacteria (PGPR) and arbuscular mycorrhizal fungi (AMF). Specifically, microbial agents like *Aspergillus*, *Arthrobacter*, and *Streptomyces* have been shown to enhance nutrient uptake, stress tolerance, and fruit quality by regulating plant hormones and maintaining cell integrity (Daşgan et al. 2008; Yılmaz et al. 2025). These microorganisms have been demonstrated to promote healthier plants and higher yields by increasing nitrogen fixation, phosphate solubility, and plant hormone production (Tzortzakis and Economakis 2008; Zaman et al. 2025). In addition to all of these, *Aspergillus* spp. has been demonstrated to promote phosphate solubilization (Tzortzakis and Economakis 2008), prevent chlorosis by increasing iron uptake under alkaline hydroponic conditions (Bozköylü and Daşgan 2010; Zaman et al. 2025), stimulate root development through hormone regulation (e.g., auxin production) (Sedaghat et al. 2017; Zaman et al. 2025). *Streptomyces* spp. have been observed to release secondary metabolites, such as actinomycin, which have been shown to suppress root pathogens, including Fusarium (Sedaghat et al. 2017; Zaman et al. 2025).

Tomatoes, whether consumed fresh, processed, or canned, represent significant economic value for growers, packers, and the global food industry. The perishable nature of tomato fruit is a significant factor contributing to substantial yield losses that occur post‐harvest. The magnitude of these losses has been found to vary between approximately 25% and 45%, depending on various factors (Pathare et al. 2023; Walubengo 2023; Roy et al. 2024). Research indicates that these issues can be mitigated by maintaining optimal storage conditions (e.g., 12°C–15°C) and selecting varieties with better cold tolerance (Toprak and Gül 2013). The utilization of beneficial fungi and bacteria, including *Aspergillus*, *Arthrobacter*, and *Streptomyces*, has been demonstrated to promote tomato growth, yield, and postharvest quality in soilless systems, concurrently mitigating cold storage challenges. These microorganisms have been demonstrated to enhance nutrient availability, stress tolerance, and shelf life through a variety of mechanisms. It has been reported that *Aspergillus* spp. species exhibit antifungal activity during cold storage, while species such as *Streptomyces* spp. provide resistance against post‐harvest fungal infections and maintain fruit firmness during storage by producing chitinases that break down fungal (Tzortzakis and Economakis 2008; Toprak and Gül 2013).

Consequently, the extension of shelf life during post‐harvest cold storage, the reduction of microbial spoilage, and the preservation of fruit quality attributes such as firmness, color, and nutritional value are of critical importance. Cold storage is a widely employed method of retarding the ripening process and reducing the rate of spoilage. While the benefits of bio‐fertilizers on plant growth are well‐documented, research on how these microorganisms influence the biochemical stability and quality changes of fruit during cold storage is still limited. This study addresses this gap by evaluating the effects of selected microbial bio‐fertilizers on the post‐harvest attributes of soilless‐grown tomatoes.

This study aimed to determine the effects of applying specific microbial biological fertilizers (*Aspergillus oryzae*, *Arthrobacter globiformis* , and *Streptomyces griseus* ) on the post‐harvest weight loss of soilless‐grown tomatoes and to identify their potential effects on changes in bioactive compounds, antioxidant stability, and post‐harvest quality during cold storage.

## 2 Material and Method

### 2.1 Experimental Time and Location

The present study was conducted at the Post‐Harvest Physiology Laboratory of the Department of Horticulture, Faculty of Agriculture, Ordu University (Ordu, Türkiye). The fieldwork for this study was conducted between 15 March and 20 July 2022, in a plastic greenhouse (39° 00′ 17.3″ N, 33° 56′ 34.9″ E, and 966 m elevation) owned by Tutku Tarım (Aksaray, Türkiye) under soilless cultivation conditions.

### 2.2 Plant and Fertilizer Materials

In this study, four‐leaf seedlings of the Cletego F1 ( *Solanum lycopersicum* L.) (Syngenta, Izmir, Türkiye) tomato variety, which is particularly suitable for soilless cultivation, were used.

The study utilized a range of microbial fertilizers, including fungal (*Aspergillus oryzae* 1 × 10^10^ CFU g^−1^) and bacterial formulations ( *Arthrobacter globiformis* 1 × 10^10^ CFU g^−1^, *Streptomyces griseus* 1 × 10^10^ CFU g^−1^). The microbial fertilizers utilized in the present study were obtained from Milicard (Türkiye). The control treatment was defined as the application where no microbial fertilizer was applied. The *Aspergillus oryzae* microbial fertilizer application was abbreviated as “AO,” the *Arthrobacter globiformis* microbial fertilizer application as “AG,” the *Streptomyces griseus* microbial fertilizer application as “SG,” and the control treatment as “C.”

### 2.3 Experiment Description

The tomato seedlings utilized in the present study were obtained from a commercial seedling supplier and cultivated in coconut fiber growing slab (100 × 20 × 5 cm). The seedlings were then planted on April 25, 2022, at equal intervals (60 cm between rows and 30 cm within rows), so that there were four plants in each growing bag. The growing bags were placed in channels within the greenhouse that were 60 m in length, 25 cm in width, and had a slope of 1.5%. Subsequent to the implementation of the planting process, the macro‐ and micronutrients utilized for fertilization purposes were meticulously prepared as stock solutions within two 2000‐l tanks, designated as A and B, respectively. The nutrient solutions to be used in the experiment (Hoagland and Arnon 1950) are given in Table 1. The stock solutions presented in Table 2 were administered at varying doses, contingent upon the growth and developmental phases of tomato plants. Temperature values recorded with data loggers (PT100, Hoogendorn, Hollanda) in the greenhouse ranged from 17°C to 28°C, while relative humidity values varied between 43% and 86%. When relative humidity values exceeded 70%, the ventilation system was used to control them. Throughout the cultivation period, a range of other maintenance, spraying and cultural procedures were applied as required. Armpit removal and pruning were executed on a weekly basis. According to the EDT (economic damage threshold) method, cultural precautions such as collecting pest leaves, placing pheromone water traps and sticky traps were carried out.

**TABLE 1:** Macro and micronutrient solutions and their ratios used in soilless cultivation.

Stock solutions	Chemical substances	The amounts
A (2000 L)	Potassium nitrate	50 kg
	Calcium nitrate	260 kg
	Nitric acid	1 L
	Iron chelate	3 L
	Potassium chloride	10 kg
	Previcur energy	600 mL
B (2000 L)	Potassium nitrate	52 kg
	Monopotassium phosphate	54 kg
	Potassium sulfate	84 kg
	Magnesium sulfate	84 kg
	Zinc sulfate	430 g
	Manganese sulfate	1000 g
	Sodium molybdate	24 g
	Copper sulfate	60 g
	Borax	570 g

**TABLE 2:** Fertilizer doses to be applied to tomatoes according to growth and development periods.

Developmental periods	mg/L				
	N	P	K	Ca	Mg
Until flowering (25–40 day)	200	50	275	250	70
After 4–5 flower clusters appear (40–60 day)	230	55	360	230	70
After 7–8 flower clusters appear (60–120 day)	220	55	470	230	60

The study utilized three distinct microorganisms (*Aspergillus oryzae*, *Arthrobacter globiformis* , *Streptomyces griseus* ) as microbial fertilizers. The application of *A. oryzae* (Milicard, MCC075 pure culture), *A. globiformis* (Milicard, MCC296 pure culture) and *S. griseus* (Milicard, MCC1973 pure culture) was conducted on a weekly basis throughout the seedling stage (1 month), utilizing 1‐l mixtures with a concentration of 10 g L^−1^. Each plant received 100 mL of the mixture, and the application was administered to six plants per treatment. During the vegetative phase following the seedling stage, the applications were made on a biweekly basis at a concentration of 10 g L^−1^. The harvesting of the fruit was conducted manually at the stage of optimal ripeness, characterized by the colouration of 30%–60% of the fruit skin, as defined by the standards established by the United States Department of Agriculture (USDA) in 1991.

### 2.4 Post‐Harvest Measurements and Observations

The study comprised four treatments: three biological fertilizers (*Aspergillus oryzae*, *Arthrobacter globiformis* , *Streptomyces griseus* ) and one control. During the process of sampling the fruits and distributing them to storage units, a systematic approach was followed to ensure the statistical independence of the results and eliminate the risk of pseudoreplication. The harvesting of the fruits was conducted at the pink ripening stage, with a total of 54 plants (18 plants per replicate) from each greenhouse plot (replicate) being sampled. The greenhouse plot configuration was established in accordance with a randomized block design. During the setup of the cold storage trial, fruits with cracked, damaged, bruised, or rotten skins were discarded, and fruits with uniform color, size, and shape were selected. A cold storage trial was established after harvesting the fruits obtained from these measurement plants. Measurements were taken during harvest (Day 0). These measurements were taken on 30 fruits for each treatment. Following the selection of fruits with uniform characteristics from the harvested crop, they were randomly distributed among a total of 36 independent plastic crates (4 treatments × 3 storage periods × 3 replicates). This approach ensured that each analysis would be represented by 3 independent replicates throughout the storage period. Each plastic crate (replicate) was defined as an independent experimental unit containing approximately 25–30 fruits, and the analyses were conducted on these independent containers during each storage period. All tomato fruits were stored in modified atmosphere packaging (MAP). The modified atmosphere bags utilized in the present study, manufactured by StePac (Xtend, Israel), are composed of LDPE, possess a thickness of 22 μm, and possess a capacity of 5 kg. These bags demonstrate a CO_2_ permeability of 2203 cc m^−2^·day·atm, as well as a water vapor permeability of 150.0 g m^−2^·day·atm at a temperature of 23°C.

Prior to this, the fruit was pre‐cooled with cold air until the flesh temperature had decreased to 10°C. The MAP bags were then sealed with rubber bands. Following a preliminary cooling phase, the tomatoes were stored for a period of 21 days at a temperature of 8°C and a relative humidity level of 90% within a cold storage facility located at the Faculty of Agriculture, Ordu University. Measurements were taken on the 7th, 14th, and 21st days during the cold storage period for the tomatoes. On each occasion that the experiment was conducted, three boxes from each treatment were selected, and each box was designated as a replicate. During the 21‐day cold storage period, the measurements and analyses specified in the methods were performed in sequence.

The weight loss percentage was measured at the commencement of the cold storage experiment (Day 0) and at each analysis interval (Days 7, 14, and 21) by weighing the fruit weights for each microbial treatment on a scale (Radwag, Poland) with a sensitivity of 0.01 g. The obtained values were expressed as a percentage by substituting them into the following formula (Özer et al. 2022).

$$\text{Weight loss}\;\left(\%\right)=\frac{\text{Harvest weight}–\text{Final weight}}{\text{Harvest weight}}\;\times \;100$$

The respiration rate was determined by measuring the fruit weight and volume. The fruits were then stored in a 2‐l airtight container (at room temperature) for 1 h. The amount of CO_2_ released by the fruits into the external environment was measured using a digital carbon dioxide analyzer (Vernier, USA), and the obtained values were expressed as mL CO_2_ kg^−1^ h^−1^ based on the weight and volume of the fruits placed in the jars (Öztürk et al. 2014).

In order to ascertain fruit firmness (%), ten fruits were used from each replicate of the experiments. The measurements were obtained using a portable digital durometer (Agrosta, France) with a flat cylindrical tip and a diameter of 4.1 mm. The tip of the durometer was gently pressed against the fruit's outer skin in a longitudinal direction, and the percentage value displayed on the screen was recorded (Öztürk and Özer 2019). A value approaching 100 indicates a high degree of hardness, while a value close to 0 indicates an excessively soft texture.

For the purpose of pH measurement, ten tomato fruits from each treatment were washed with distilled water, then chopped in a kitchen food processor (Model No. AR1055‐O Prochopp Plus Arzum, Türkiye) and strained. The resulting fruit juice samples were then utilized to ascertain the pH, titratable acidity, total soluble solids, and vitamin C content of the fruit juices. The pH of the fruit juice was determined using a pH meter (WTW 3110 Set 2 pH Meter, Germany) (Karaçalı 2009).

The measurement of titratable acidity (TA) was conducted by extracting 10 mL of juice from the harvested tomato fruits and adding 10 mL of distilled water. The mixture was subjected to the addition of a 0.1 N NaOH (sodium hydroxide) solution until the pH reached 8.2. The titratable acidity was determined on the basis of the amount of NaOH consumed during the titration process. This was expressed as grams of citric acid per 100 mL (Özer et al. 2022). In order to ascertain the total soluble solids (SSC) values, SSC content in the fruit juice samples from each replicate was measured using a portable digital refractometer (Atago PAL‐1, USA) and expressed as a percentage (Öztürk and Özer 2019). The analysis of vitamin C content was conducted utilizing the Reflectoquant Plus 10 device (Merck RQflexplus 10, Türkiye), in accordance with the protocol delineated by Uğur et al. (2022). For the analysis, 0.5 mL of the extracted fruit juice was mixed with 0.5% oxalic acid and diluted to 5 mL. An ascorbic acid test kit (Catalog No. 116981, Merck, Germany) was immersed in the solution for a period of 2 s, oxidized for 8 s, and then placed in the Reflectoquant device. Subsequent to the application of the test kit, a five‐second waiting period was observed before the results were read and determined in mg 100 g^−1^.

### 2.5 Sample Preparation for Bioactive Compound Analyses

Following a thorough cleansing with distilled water, the fruits were meticulously sliced and homogenized using a kitchen‐type blender (Model No. AR1055‐O Prochopp Plus Arzum, Türkiye). The resulting homogenates were transferred to numbered 50 mL Falcon tubes and stored at −20°C for biochemical analysis. At the time of analysis, the samples were removed from the freezer and thawed at room temperature (approximately 23°C–24°C). Approximately 1 g of fruit sample was taken from the experimental samples, and methanol (CAS No: 67–56‐1, Merck, Germany) was added to the samples. The mixture was transferred to new Falcon tubes and stored in a refrigerator (4°C) for a minimum of 48 h prior to analysis of phenolic, flavonoid, and antioxidant activity.

The total phenolic content was determined using the Folin–Ciocalteu method, as described by Peksen et al. (2023). Following the amalgamation of 1000 μL of the fresh fruit extract with 3.6 mL of pure distilled water, a solution was prepared by the subsequent addition of 100 μL of Folin–Ciocalteu reagent and 100 μL of 2% sodium carbonate (Na_2_CO_3_). The solution was then subjected to incubation in conditions of darkness for a period of 2 h. Following this, the absorptivity of the blue‐colored solution was measured at a wavelength of 760 nm, using a UV–vis spectrophotometer (Shimadzu, UV‐1280, Japan). The results were calculated as gallic acid equivalents and expressed in grams per kilogram of fresh weight (g GAE kg^−1^).

The method described by Ozturk et al. (2024) was utilized to determine total flavonoids. Following the addition of 3.3 mL of methanol to 1000 μL of fruit extract, 100 μL of a 10% aluminium nitrate [Al(NO_3_)_3_] and 0.1 M ammonium acetate (NH_4_CH_3_CO_2_) solution were added. Following an incubation period of 40 min in conditions of darkness and ambient temperature, the samples were measured at a wavelength of 415 nm by means of a UV–vis spectrophotometer. The results were expressed as milligrams of quercetin equivalent (QE) per 100 g of fresh weight.

The DPPH antioxidant activity of the tomato fruit extract was evaluated according to the protocol of the Blois (1958) method, as modified by Öztürk et al. (2016). The DPPH solution was utilized as the source of free radicals. Stock solutions were prepared at varying concentrations, with 0.5 mL of each solution mixed with 0.1 mM DPPH in ethanol. The total solution volume was adjusted to 3 mL, and the solution was mixed and left to stand for 30 min under dark room temperature conditions. A series of sample readings were obtained at a wavelength of 517 nm, employing a UV–vis spectrophotometer. The resulting data were reported in millimoles of Troloks equivalent per kilogram (mmol TE kg^−1^). For the ferric reducing antioxidant power (FRAP) assay, 120 μL of extract was mixed with 0.2 M phosphate buffer (pH 6.6) to a final volume of 1.25 mL. Subsequently, 1.25 mL of a 1% potassium ferricyanide (K₃Fe(CN)₆) solution was added to the mixture and incubated at 50°C. Following the incubation process, 1.25 mL of 10% trichloroacetic acid (TCA) and 0.25 mL of 0.1% iron chloride (FeCl3) were added to the solution. The samples were measured at a wavelength of 700 nm using a UV–vis spectrophotometer, and the results were expressed in millimoles of Troloks equivalent per kilogram (mmol TE kg^−1^) following the protocols described by Peksen et al. (2023).

### 2.6 Statistical Analysis

Tomato fruits obtained from four different treatments, including three different biological fertilizers (*Aspergillus oryzae*, *Arthrobacter globiformis* , *Streptomyces griseus* ) and a control group, were harvested at the pink ripening stage. A cold storage trial was established after harvesting the fruits obtained from these measurement plants. Measurements were taken during harvest (Day 0). These measurements were taken on 30 fruits for each treatment. Then the post‐harvest study was started.

A total of 36 plastic crates containing tomatoes (4 treatments × 3 storage periods × 3 repetitions) were placed in the cold storage facility for the treatments. The experiment followed a completely randomized design with three biological replicates per treatment. The statistical analysis software SPSS (Version 15.0, New York, USA) was utilized to evaluate the data obtained from the study. Significant differences among treatments were determined using one‐way ANOVA followed by Tukey's multiple comparison test at *p* < 0.05.

## 3 Results

### 3.1 Weight Loss, Respiration Rate and Fruit Firmness

Bio‐fertilizers (*Aspergillus oryzae*, *Arthrobacter globiformis* , and *Streptomyces griseus* ) were found to have significant effects (*p* < 0.05) on post‐harvest weight loss, respiration rate, and fruit firmness of tomatoes grown in soilless conditions. The results concerning weight loss, respiration rate, and fruit firmness in tomatoes treated with various microbial fertilizers and stored in cold conditions after harvest. Examining the weight loss values revealed that the AO treatment differed significantly from the others by day 7. The highest weight loss (0.11%) was observed in the AO treatment, while the lowest weight loss (0.07%) was observed in the AG treatment. There were no statistically significant differences in the average weight loss values obtained on days 14 and 21 among the treatments. Although the weight loss characteristic became evident on day 7, no significant differences were observed among the treatments during other storage periods (Table 3).

**TABLE 3:** The effect of biological fertilization with pure cultures of *Aspergillus oryzae*, *Arthrobacter globiformis* , and *Streptomyces griseus* on tomato weight loss, respiration rate, and fruit firmness during cold storage.

Treatments	Weight loss (%)	Respiration rate (mL CO_2_ kg^−1^ h^−1^)	Firmness (%)
Harvest			
C	0	16.85 c	5.50
AO	0	21.30 a*	5.30
AG	0	16.06 d	4.82
SG	0	19.14 b	5.02
7 days at 8°C			
C	0.10 ab	4.56 c	4.78
AO	0.11 a*	4.77 c	3.66
AG	0.07 b	8.24 a*	4.60
SG	0.08 ab	6.03 b	3.48
14 days at 8°C			
C	0.14	4.28 d	4.52
AO	0.16	6.68 b	5.00
AG	0.13	7.51 a*	4.98
SG	0.13	6.10 c	4.28
21 days at 8°C			
C	0.19	5.96 a*	1.90
AO	0.20	4.64 b	2.02
AG	0.18	3.58 c	2.14
SG	0.18	4.66 b	1.98

*Note:* Different letters are significantly different at *p ≤ 0.05.

Statistical analysis revealed significant variations in respiration rates among the treatments on Day 0. The respiration rate was found to be the highest in tomato fruits treated with AO (21.30 mL CO_2_ kg^−1^ h^−1^). The lowest respiration rate (16.85 mL CO_2_ kg^−1^ h^−1^) was observed in the AG treatment, while the control treatment yielded values close to this. On the seventh day, the AG treatment was found to have the highest respiration rate (8.24 mL CO_2_ kg^−1^ h^−1^), and a significant difference was observed when compared to the other treatments. On the seventh day, the C and AO treatments were found to be statistically indistinguishable. On the fourteenth day, the respiration rate was once more highest in the AG treatment (7.51 mL CO_2_ kg^−1^ h^−1^), and significant differences were observed among the treatments (*p* < 0.05). The control treatment was once again observed to be in the lowest group, as was the case on day 7. On the 21st day, the highest respiration rate (5.96 mL CO_2_ kg^−1^ h^−1^) was observed in tomatoes from the control treatment, while the AO and SG treatments were in the same group, and the AG treatment had the lowest respiration rate (3.58 mL CO_2_ kg^−1^ h^−1^). On the basis of the entire storage period, it was determined that the AO treatment generally increased the respiration rate. In contrast, the AG and SG treatments exhibited variable effects at different storage durations.

Subsequent to the examination of fruit firmness, it was determined that there were no statistically significant differences between the treatments at harvest (Day 0) or throughout the storage period. It was observed that fruit flesh firmness decreased in proportion to the length of the storage period. On day 0, the highest recorded firmness value of 5.50% was observed in the control (C) group. Subsequent to this, the AO (5.30%), SG (5.02%), and AG (4.82%) treatments were administered, respectively. Firmness loss was observed in all groups on day 7. While the control group exhibited the highest value at 4.78%, the lowest firmness was recorded at 3.48% in the SG treatment. By day 14, the AO (5.00%) and AG (4.98%) treatments began to demonstrate a superior capacity to preserve the fruit tissue, exhibiting higher firmness values in comparison to the control group (4.52%). By day 21, a significant decrease in firmness was observed in all groups at the end of storage. However, the microbial and organic‐based treatments AG (2.14%) and AO (2.02%) were successful in maintaining firmer fruit flesh compared to the control group (1.90%).

### 3.2 pH, Acidity, Solube Solid Content, and Vitamin C

Microbial fertilizer applications were found to have significant effects on the acidity, total soluble solids, and vitamin C content of tomato fruits during the post‐harvest storage period. While the effects of the applications on the pH values of the fruit were not determined, acidity, total soluble solids, and vitamin C values were unaffected on the 21st day. The pH values ranged from 4.3 to 4.4 on Day 0 and from 4.20 to 4.61 on Day 21. The pH values were similar across all treatments and were not significantly affected by storage duration (Table 4).

**TABLE 4:** The effect of biological fertilization with pure cultures of *Aspergillus oryzae*, *Arthrobacter globiformis* , and *Streptomyces griseus* on tomato on pH, acidity, soluble solid content, and Vitamin C of tomato fruit during cold storage.

Treatments	pH	Acidity (g citric acid 100 mL^−1^)	SSC (%)	Vitamin C (mg 100 g^−1^)
Harvest				
C	4.30	0.21 b	4.25 c	23.10
AO	4.30	0.28 a*	4.85 a*	11.80
AG	4.35	0.22 b	4.55 b	13.80
SG	4.40	0.28 a	4.40 bc	16.30
7 days at 8°C				
C	4.45	0.24 ab	5.90 a*	14.60 ab
AO	4.50	0.26 a*	4.80 b	13.80 b
AG	4.50	0.17 b	4.50 b	12.70 b
SG	4.47	0.23 ab	4.80 b	15.80 a*
14 days at 8°C				
C	4.68	0.20 b	5.20 a	14.70 a
AO	4.72	0.21 ab	4.60 b	11.90 b
AG	4.60	0.23 a	5.10 a*	12.00 b
SG	4.72	0.19 b	4.60 b	15.30 a*
21 days at 8°C				
C	4.20	0.15	4.70	17.70
AO	4.55	0.21	4.40	21.00
AG	4.58	0.20	4.90	23.60
SG	4.61	0.22	4.90	20.40

*Note:* Different letters are significantly different at *p ≤ 0.05.

When acidity levels are examined, the AO and SG treatments exhibited the highest levels on Day 0, with an acidity value of 0.28 g citric acid per 100 mL. On the seventh day, the AO treatment exhibited the highest acidity, while the lowest acidity value was observed in the AG treatment. On the fourteenth day, the AG treatment exhibited the highest acidity levels (0.23 g citric acid per 100 mL‐1), while the AO, SG, and C treatments yielded comparable values. On the 21st day, no statistically significant difference was observed in the means. The highest acidity value was determined in the SG treatment, and the lowest acidity value was observed in the control treatment. The interpretation of the acidity results across all storage periods revealed that the AO treatment generally exhibited higher acidity values. Statistically significant differences (*p* < 0.05) were observed among the treatments on days 0, 7, and 14, but no significant difference was found on day 21.

On day 0, the AO treatment exhibited the highest value (4.85%), while the control treatment demonstrated the lowest (4.25%). On the seventh day, the control treatment exhibited the highest value (5.90%), while microbial fertilizer applications demonstrated a significant reduction in soluble solid content (4.50%–4.80%). On the fourteenth day, the control and AO treatments exhibited the highest values, respectively (5.20% and 5.10%). No significant differences were observed among the treatments on the 21st day. The control treatment demonstrated elevated SSC values on the 7th and 14th days.

Levels of ascorbic acid were found to be statistically insignificant on days 0 and 21, but statistically significant on days 7 and 14. On day 0, the Control group exhibited the highest value (23.10 mg 100 g^−1^). The application of biofertilisers resulted in a substantial reduction in the Vitamin C content (AO‐11.80, AG‐13.80, SG‐16.30). On the seventh day, the SG and control treatments exhibited the highest values, respectively (15.80 mg 100 g^−1^ and 14.60 mg 100 g^−1^). On the fourteenth day, the SG (15.30 mg 100 g^−1^) and control (14.70 mg 100 g^−1^) treatments exhibited statistically higher vitamin C contents in comparison to the remaining treatments. On day 21, the highest vitamin C content was obtained from the AG treatment (23.60 mg 100 g^−1^), while the lowest vitamin C content was analyzed in the control treatment (17.70 mg 100 g^−1^).

### 3.3 Total Phenolics, Total Flavonoids and Total Antioxidant Activity

Bio‐fertilizers (*Aspergillus oryzae*, *Arthrobacter globiformis* , and *Streptomyces griseus* ) were found to have significant effects (*p* < 0.05) on post‐harvest total phenolics, total flavonoids, DPPH and FRAP of tomatoes grown in soilless conditions (Table 5). On day 0, the highest values were obtained from the SG, AG, and AO treatments (12.19, 11.48, and 10.27 mg GAE 100 g^−1^), respectively; these values were statistically significantly higher than those of the control group (*p* < 0.05). On the seventh day, the mean total phenolic content values for the treatments were found to be statistically indistinguishable from one another. During this storage period, the control group also exhibited lower values compared to the other treatments. On the fourteenth day, a significant difference was observed in the values for the AO treatment in comparison to the other treatments. The control group yielded the lowest values. On the 21st day, the AG treatment was found to be particularly noteworthy. The lowest values were observed in the SG treatment. A generalized increase in the total phenolic compounds was observed as the storage period increased. While microbial fertilizer treatments yielded high values throughout the storage period, the control group's tomatoes exhibited the lowest averages, except on the 21st day.

**TABLE 5:** The effect of biological fertilization with pure cultures of *Aspergillus oryzae*, *Arthrobacter globiformis* , and *Streptomyces griseus* on tomato total phenolics, total flavonoids, and antioxidant activity (DPPH and FRAP) of tomato fruit during cold storage.

Treatments	Total phenolics (mg GAE 100 g^−1^)	Total flavonoids (mg QE 100 g^−1^)	DPPH (mmol TE 100 g^−1^)	FRAP (mmol TE 100 g^−1^)
Harvest				
C	5.58 b	4.08 b	4.88 b	14.67 ab
AO	11.48 a	6.57 a	9.54 b	16.80 a*
AG	10.27 a	7.61 a*	10.49 a*	13.02 b
SG	12.19 a*	3.05 b	9.08 b	16.80 a*
7 days at 8°C				
C	10.67	2.36 c	12.72 a*	17.34 a*
AO	12.06	8.63 ab	9.04 c	17.08 a
AG	13.42	10.09 a*	6.45 d	12.46 b
SG	13.22	7.83 b	10.66 b	16.30 a
14 days at 8°C				
C	9.74 c	5.52 a	9.27 b	14.34 ab
AO	16.21 a*	2.21 b	11.15 a*	16.24 a
AG	14.06 ab	3.52 b	9.64 b	11.35 b
SG	11.93 bc	6.68 a*	8.34 c	16.26 a*
21 days at 8°C				
C	13.47 ab	8.24 c	9.71 b	20.04 a*
AO	13.76 ab	10.88 b	11.72 a*	12.19 b
AG	16.43 a*	13.57 a*	8.33 bc	18.97 a
SG	10.76 b	3.80 d	7.23 c	19.24 a

* Different letters are significantly different at *p* ≤ 0.05.

As demonstrated in Table 5, the highest total flavonoid values were observed on days 0 and 7 in the AG and AO treatments, respectively. The application of AG treatment resulted in substantial variations in both storage periods. However, on day 14, the SG (6.68 mg QE 100 g^−1^) and C (5.52 mg QE 100 g^−1^) treatments yielded the highest values, while the AG and AO treatments had low flavonoid content during this storage period. By the conclusion of day 21, a number of significant differences were observed among the treatments, with the highest value (13.57 mg QE 100 g^−1^) once again detected in the AG treatment. This treatment was followed, in order, by AO (10.88 mg QE 100 g^−1^), C (8.24 mg QE 100 g^−1^), and SG (3.80 mg QE 100 g^−1^). In general, the application of AG resulted in the highest values when storage periods were taken into consideration. While the AG treatment demonstrated notable resilience during the extended storage period, the SG treatment exhibited the poorest performance.

The application of AG has been identified as one of the most effective methods for increasing the total flavonoid content in tomatoes during storage. The study found that the flavonoid content in fruits treated with AG was 7.61 mg QE 100 g^−1^ at harvest, reaching 10.09 mg QE 100 g^−1^ on the 7th day of storage and 13.57 mg QE 100 g^−1^ on the 21st day, demonstrating the highest values throughout the entire storage period.

On day 0, the DPPH antioxidant activity was examined, and the highest value, 10.49 mmol TE 100 g^−1^, was measured in the AG treatment. The other treatments (AO: 9.54 mmol TE 100 g^−1^, SG: 9.08 mmol TE 100 g^−1^) exhibited higher initial antioxidant capacity compared to the control (4.88 mmol TE 100 g^−1^). On the seventh day, the control group (C) demonstrated the highest value of 12.72 mmol TE 100 g^−1^, while the AG treatment exhibited the lowest level of 6.45 mmol TE 100 g^−1^. On the fourteenth day, the AO treatment was found to be most significant, with a value of 11.15 mmol TE 100 g^−1^. On the final day of storage, Day 21, the highest radical scavenging activity was recorded in the AO treatment at 11.72 mmol TE 100 g^−1^. This was followed, in order, by the control (9.71 mmol TE 100 g^−1^), AG (8.33 mmol TE 100 g^−1^), and SG (7.23 mmol TE 100 g^−1^) (Table 5).

Upon examination of the FRAP values, it was determined that the highest values were measured in the AO and SG treatments at 16.80 mmol TE 100 g^−1^ at harvest. On the seventh day, the control group (C) demonstrated the highest antioxidant capacity, with a value of 17.34 mmol TE 100 g^−1^. In comparison, the AO (17.08 mmol TE 100 g^−1^) and SG (16.30 mmol TE 100 g^−1^) treatments exhibited values that were comparable to those of the C group. On the fourteenth day, the highest recorded value was 16.26 mmol TE 100 g^−1^ in the SG treatment, while the lowest value was 11.35 mmol TE 100 g^−1^ in the AG treatment. On the final day of storage (Day 21), the control (C) group demonstrated the highest FRAP capacity at 20.04 mmol TE 100 g^−1^, while among the microbial treatments, SG (19.24 mmol TE 100 g^−1^) and AG (18.97 mmol TE 100 g^−1^) exhibited comparable performance to the control. It was observed that the antioxidant properties of biofertilizers in cold‐stored tomato fruits were effective in increasing antioxidant capacity at harvest (particularly AG on DPPH, and AO and SG on FRAP). Fluctuations in values were observed throughout the storage process; by the conclusion of the storage period, AO yielded superior results for DPPH, while the control and SG treatments yielded superior results for FRAP.

## 4 Discussion

Microbial fertilization, particularly the use of arbuscular mycorrhizal fungi (AMF) and plant growth‐promoting rhizobacteria (PGPR), is a key biological strategy for extending the shelf life of tomatoes after harvest and reducing physiological weight loss (Violante and Pérez 2022; Torres‐Solórzano et al. 2025). It has been demonstrated that plants cultivated with microbial fertilization exhibit enhanced water use efficiency and a fortified cuticle layer (Carillo et al. 2020; Gashash et al. 2022). In accordance with this physiological condition, the present study found that AG and SG applications reduced weight loss on day 7. In the context of storage studies on tomatoes, the impact of microbial fertilization on weight loss has been ascribed to various factors. These include the restriction of water loss and transpiration (Özer et al. 2022), the maintenance of mineral balance (Suebrasri et al. 2023; Adekiya et al. 2025; Torres‐Solórzano et al. 2025), the alleviation of oxidative stress (Soussani et al. 2023), the preservation of membrane integrity (Soussani et al. 2023), and the regulation of respiration rate (Adekiya et al. 2025).

As demonstrated in the study by Gonfa et al. (2021), biofertilization exerts a regulatory and inhibitory effect on the post‐harvest respiration rate of tomatoes. The process of microbial fertilization has been shown to physiologically prepare the plant prior to harvest. This preparation has been shown to enhance the activity of enzymes (SOD, CAT, POD, APX) that play a critical role in scavenging reactive oxygen species (ROS) that arise during storage, particularly in response to cold stress (Rosales et al. 2009; Javanmardi et al. 2013; Khazaei et al. 2026). Furthermore, it has been demonstrated that cell integrity is maintained through microbial inoculation, thereby preventing abnormal respiration fluctuations triggered by tissue damage (Wei et al. 2025). The observation of the lowest respiration rate in the AG treatment on day 21 can be considered a result of this mechanism. During the same storage period, the control treatment exhibited the highest respiration rate, indicating that microbial inoculation slows down the respiration rate in tomato fruits during cold storage.

Firmness is a very important parameter in determining fruit quality, ripeness level, and transportability. Similarly to our study, it has been observed that some microorganisms increase calcium accumulation in the fruit peel (fruit peel and flesh), thereby increasing the firmness of the fruit flesh (Carillo et al. 2020; Suebrasri et al. 2023; Adekiya et al. 2025). Moreover, it has been documented that microbial applications have the capacity to retard the activity of enzymes responsible for the breakdown of cell walls, with a particular emphasis on PG (Yahia et al. 2005; Oladipo et al. 2025). The application of microbial fertilizers has been demonstrated to stimulate the antioxidant defense system (SOD, CAT, and POD enzymes) of plants. This process assists in maintaining the cells' internal turgor pressure, thereby preserving the fruit's crisp texture. This is achieved by preventing damage to cell membranes caused by oxidative stress (Soussani et al. 2023; Wei et al. 2025). In tomatoes cultivated in soilless culture with microbial fertilization using *Streptomyces griseus* and *Arthrobacter globiformis* , fruits that were comparable to or more resilient than the control were obtained, thereby indicating that market value was maintained (Yılmaz et al. 2025).

Our current results, similar to previous studies, show a decrease in fruit firmness during storage (Öztürk and Özer 2019; Özer et al. 2022; Ozturk et al. 2024; Saka et al. 2025). However, unlike others, in our study, the application of AG limited this decrease. This is because the highest firmness values were measured in fruits treated with AG at the final stage of storage (day 21). This effect may be due to the protective effect of microbial fertilization on the cell wall, as mentioned by Zaman et al. (2025). The fact that AO application also showed superiority compared to the control group demonstrates the superiority of biofertilizer applications over the control in any case. In conclusion, we can say that microbial fertilization acts as a biological shield, protecting the firmness of the fruit flesh and delaying the softening of tomatoes during post‐harvest storage.

Harvested fresh produce undergoes physiological changes after harvest. This process is further complicated by the presence of microorganisms that cause decay, wilting, and degradation of essential nutrients (Ncama et al. 2018; Papoutsis and Edelenbos 2021; Zaman et al. 2025). While current conventional preservation methods such as cold storage, modified atmosphere packaging (MAP), and ethylene inhibitors are effective to some extent, they have limitations (Chiabrando and Giacalone 2009). Understanding the interaction between physiological processes and microbial growth is crucial for developing strategies to extend the shelf life of perishable produce (Mari et al. 2016; Nasr and Soliman 2023; Zaman et al. 2025). Microbial interventions can extend shelf life by reducing spoilage, offering a sustainable and effective approach to post‐harvest management. In this way, they preserve the quality of horticultural produce. Beneficial microorganisms such as bacteria, fungi, and yeasts are reported to play crucial roles in suppressing pathogenic microbes, regulating ripening through ethylene modulation, and enhancing the nutritional value of stored products (Zaman et al. 2025). Similar findings were observed in our study.

In a study by Bozköylü and Daşgan (2010), the pH values in groups receiving chemical and organic fertilization were found to range between 4.3 and 4.4, and the difference between them was deemed to be statistically insignificant. In a study on bell peppers by Dasgan et al. (2023), it was observed that the application of mycorrhizal fungi did not result in a statistically significant difference in pH levels. However, due to the climacteric nature of tomatoes, a slight tendency toward a periodic increase in pH may be observed as ripening progresses, attributed to the consumption of organic acids (citric and malic acid) during respiration (Mujtaba and Masud 2014; Peralta‐Ruiz et al. 2020). As established in preceding studies, the impact of cultivation systems and fertilization methods on pH levels has been shown to be statistically non‐significant.

Findings are consistent with the extant literature, which indicates that microbial inoculants have the capacity to influence the organic acid content of fruits by modulating metabolic pathways, enzyme activities, and stress responses. For instance, it has been demonstrated that arbuscular mycorrhizal fungi and PGPRs impact acid metabolism by enhancing nutrient uptake and hormonal regulation, thereby influencing fruit acidity and flavor stability during storage (Tzortzakis and Economakis 2008; Daşgan et al. 2008). The consistently high acidity levels observed in AO‐treated fruits may be attributable to its established function in preserving organic acids over an extended period by impeding the process of overripening and ethylene biosynthesis (Daşgan et al. 2008; Toprak and Gül 2013). Furthermore, changes in acidity may be indicative of microbial effects on respiratory metabolism and organic acid turnover under cold stress conditions (Rosales et al. 2009).

The findings of this study are consistent with the extant literature, which suggests that microbial applications can influence SSC by modulating fruit metabolism, nutrient uptake, and stress responses. The application of microbial fertilization, predominantly with fungi and beneficial bacteria, has been demonstrated to enhance nutrient availability and metabolic activity, thereby promoting sugar accumulation and soluble solids in fruits (Tzortzakis and Economakis 2008; Daşgan et al. 2008). The elevated SSC levels observed in fruits treated with AO during the harvest period suggest an augmentation in carbohydrate metabolism or sugar translocation. This finding is in accordance with the documented reports that fungal inoculants facilitate photosynthetic distribution and sugar synthesis (Carillo et al. 2020). Higher SSC levels in the control group at 7 and 14 days may be indicative of the natural ripening process without microbial treatments. However, biofertiliser treatments have been observed to delay or stabilize metabolic changes, which could contribute to a slowing of SSC fluctuations during storage. As rapid SSC changes are generally linked to overripening or spoilage, this stabilization may be advantageous for maintaining fruit quality and shelf life (Kyriacou and Rouphael 2018). Maboko et al. (2013) found that inoculating soil‐less‐grown tomatoes with mycorrhizae improved fruit quality and, in particular, increased total soluble solids. It is evident that microbial fertilization exerts a significant influence on the dynamics of soluble solids content (SSC) in tomatoes. This influence is manifested through its impact on physiological and biochemical processes associated with sugar metabolism and stress tolerance. Consequently, this influence contributes to the enhancement of postharvest quality. These effects underscore the potential of microbial applications as sustainable tools for regulating fruit aroma and texture during cold storage, thereby providing further evidence regarding the microbial enhancement of the fruit's nutritional and sensory characteristics (Frusciante et al. 2007; Carillo et al. 2019; Zaman et al. 2025).

Our findings are consistent with the extant literature, which indicates that microbial inoculants can modulate vitamin C content through complex physiological and biochemical mechanisms. It has been established that microbial treatments, notably those employing plant growth‐promoting rhizobacteria (PGPR) and fungi, exert an influence on antioxidant metabolism and stress responses in plants (Ordookhani et al. 2010; Soussani et al. 2023). The initial decrease in vitamin C content in microbial applications may be attributable to increased metabolic activity or the redistribution of antioxidants during the fruit's early developmental stage or at harvest. However, the enhanced preservation and increased levels of vitamin C in tomato fruits treated with AG during subsequent storage stages suggest that *Arthrobacter globiformis* mitigates the oxidative degradation of vitamin C during cold stress by activating antioxidant defense enzymes (e.g., SOD, CAT, POD) (Rosales et al. 2009; Wei et al. 2025).

Conversely, microbial inoculants have been demonstrated to enhance cell membrane integrity and curtail the accumulation of reactive oxygen species (ROS), thereby contributing to the preservation of vitamin C stability in stored fruits (Soussani et al. 2023). This finding is consistent with the findings of studies reporting that biofertilizer improves postharvest fruit quality by delaying nutrient degradation and extending shelf life (Frusciante et al. 2007; Carillo et al. 2019). The fluctuations in vitamin C levels during biofertilizer applications and storage periods highlight the differing temporal effects of microbial species on antioxidant metabolism and reinforce the importance of selecting microbial inoculations appropriate for specific postharvest quality objectives. The observed vitamin C dynamics provide support for the role of biofertilizer, particularly with *Arthrobacter globiformis* , in preserving the nutritional quality of tomatoes during cold storage by modulating antioxidant systems and reducing oxidative damage.

The results obtained demonstrate that the application of biofertilisers and the duration of their storage have a considerable impact on the levels of phenolic and flavonoid compounds in soilless tomato cultivation. It has been determined that PGPR species, including Pseudomonas and Azotobacter, enhance the nutritional status of the fruit and promote the production of antioxidant molecules (Ordookhani et al. 2010; González Rodríguez et al. 2018; Inculet et al. 2019; Hernández et al. 2020; Katsenios et al. 2021). In the present study, it was observed that the application of AG ( *Arthrobacter globiformis* ) specifically resulted in the highest total phenolic content (16.43 mg GAE 100 g^−1^) at the end of 21 days of cold storage. Conversely, microbial inoculations have been shown to impede the consumption of phenolic compounds by virtue of their capacity to stimulate antioxidant systems (SOD, CAT, POD) that function by scavenging reactive oxygen species (ROS) (Soussani et al. 2023; Wei et al. 2025). Furthermore, it has been demonstrated that tomatoes cultivated using organic‐microbial methods exhibit significantly higher levels of phenolic compounds compared to conventionally cultivated tomatoes after 21 days of storage at 8°C (Özer et al. 2022). The process of microbial fertilization has been shown to prepare tomatoes for the storage process at the cellular level (Al‐Saif et al. 2024). These practices have been shown to increase the amount of phenolic compounds at harvest, whilst also preventing the breakdown of these compounds during storage. This, in turn, has been demonstrated to extend both the nutritional value and shelf life of the fruit (Frusciante et al. 2007; Kyriacou and Rouphael 2018; Carillo et al. 2019).

The application of microbial inoculants, which include microorganisms of the Arthrobacter genus, has been demonstrated to activate the secondary metabolism of plants, thereby promoting the synthesis of phenolic compounds and flavonoids (Saia et al. 2019; Wang et al. 2023). It can be posited that the AG treatment exhibited a high degree of efficacy in preserving and enhancing the bioactive components of tomatoes during storage (at 8°C), thereby contributing to the maintenance of both the nutritional quality and market value of the fruit.

The findings of this study are consistent with the observations reported by Ordookhani et al. (2010) and Soussani et al. (2023) on the capacity of PGPR and AMF to enhance antioxidant activity. The study demonstrates that PGPR and fungal fertilizer stabilize phenolic compounds by activating enzymatic antioxidant defenses (SOD, CAT, POD) and reducing oxidative stress during storage. The initial increase observed by AG and the sustained antioxidant activity by AO indicate different microbial modulation in the scavenging of reactive oxygen species (ROS). This finding is further corroborated by the works of Wei et al. (2025) and Rosales et al. (2009).

With regard to the FRAP antioxidant capacity, the elevated initial values observed in the AO and SG treatments, and the superior preservation of FRAP values by the SG and control groups during storage, are consistent with findings that biofertilizers enhance ferric reduction capacity through phenolic stabilization and enzymatic activity (Peksen et al. 2023). The decline in FRAP values in the AG group during storage indicates the variability of microbial activity over time. This finding is consistent with the complexity observed by Kyriacou and Rouphael (2018) in relation to microbial effects on secondary metabolism and antioxidant dynamics. The total phenolic and flavonoid data provide further validation of the antioxidant findings. The superior performance of AG in preserving and enhancing these bioactive compounds during storage supports the role of microbial inoculants in activating secondary metabolite pathways, as reported by Saia et al. (2019) and Wang et al. (2023). This increased phenolic content has been demonstrated to be associated with improved nutritional quality and shelf life, a finding that is consistent with the observations reported by Frusciante et al. (2007) and Carillo et al. (2019).

The results obtained from this study demonstrate that biofertilizers, particularly with AO, AG, and SG, exert a favorable influence on antioxidant systems, phenolic stability, and fruit quality during cold storage. This finding aligns with the findings of a substantial body of literature that has identified biofertilizers as a sustainable method for enhancing the quality and stress tolerance of tomatoes post‐harvest (Daşgan et al. 2008; Tzortzakis and Economakis 2008; Torres‐Solórzano et al. 2025). The differing temporal effects of each microbial treatment highlight the necessity of selecting appropriate inoculants based on the desired harvest and storage outcomes.

Changes occurring during microbial interactions under post‐harvest conditions help to reveal the effects of beneficial microbes on plant biochemistry. During storage, horticultural products undergo a number of metabolic changes such as sugar degradation, organic acid production, and accumulation of secondary metabolites such as phenolics and flavonoids. The application of beneficial microbes has been shown to stabilize the levels of antioxidants, vitamins, and phenolic compounds (Mosier et al. 2013; De Bruyn et al. 2016; Tomás‐Barberán et al. 2000; Brizzolara et al. 2020; Li et al. 2023; Zaman et al. 2025). Some microorganisms can stimulate the synthesis of bioactive compounds that contribute to the overall quality of fruits and vegetables. In light of all this, it is reported that storage conditions and microbial processes can be optimized to extend shelf life (Brader et al. 2014; Pinu 2016; Chamkhi et al. 2021; Ling et al. 2023; Zaman et al. 2025). In parallel with these studies, our study determined that exposure to different microorganisms contributed to the post‐harvest metabolism of tomato fruits and extended their storage period.

## 5 Conclusion

The study concludes that bio‐fertilization with Aspergillus oryzae (AO), *Arthrobacter globiformis* (AG), and *Streptomyces griseus* (SG) significantly enhances the post‐harvest quality and antioxidant stability of soilless‐grown tomatoes during cold storage. Specifically, the application of AG led to a substantial increase in total phenolics, flavonoids, and antioxidant capacity at harvest, while also effectively preserving vitamin C levels by the end of the storage period. AO and SG were found to increase the ferric reducing antioxidant power (FRAP), with SG demonstrating a better ability to maintain this capacity during storage. Furthermore, these microbial treatments improved the physical shelf life by reducing weight loss and respiration rates while maintaining fruit firmness. These positive outcomes are attributed to the microbial activation of enzymatic antioxidant defenses, which mitigate oxidative stress and delay the aging process. Ultimately, microbial fertilization is a sustainable strategy for preserving the nutritional value and extending the shelf life of tomatoes, with each species offering unique temporal benefits that can be tailored to specific post‐harvest objectives.

## Author Contributions

**Sedanur Yılmaz:** investigation, data curation, visualization. **Andaç Kutay Saka:** writing – original draft, data curation, investigation, visualization. **Aslıhan Çilingir Tütüncü:** investigation, data curation, visualization. **Mehtap Özbakır Özer:** writing – review and editing, data curation, investigation, visualization. **Harun Özer:** investigation, visualization, methodology, data curation, supervision, writing – original draft.

## Funding

The authors have nothing to report.

## Ethics Statement

The authors have nothing to report.

## Consent

All authors agree to publish the final manuscript.

## Conflicts of Interest

The authors declare no conflicts of interest.

## Data Availability

The findings of this study are supported by all the data included in the article.
